# Traditional knowledge regarding edible insects in Burkina Faso

**DOI:** 10.1186/s13002-018-0258-z

**Published:** 2018-09-14

**Authors:** Aminata Séré, Adjima Bougma, Judicaël Thomas Ouilly, Mamadou Traoré, Hassane Sangaré, Anne Mette Lykke, Amadé Ouédraogo, Olivier Gnankiné, Imaël Henri Nestor Bassolé

**Affiliations:** 1 Département de Biochimie Microbiologie, Université Ouaga I Professeur Joseph KI-Zerbo, 03 BP 7021, Ouagadougou 03, Burkina Faso; 2 Département Productions Forestières, Institut de l’Environnement et de Recherches Agricoles (INERA), 03 BP 7047, Ouagadougou 03, Burkina Faso; 3 Department of Bioscience, Aarhus University, Vejlsøvej 25, 8600 Silkeborg, Denmark; 4 Département de biologie et physiologie végétale, Université Ouaga I Professeur Joseph KI-Zerbo, 03 BP 7021, Ouagadougou 03, Burkina Faso; 5 Département de biologie et physiologie animales, Université Ouaga I Professeur Joseph KI-Zerbo, 03 BP 7021, Ouagadougou 03, Burkina Faso

**Keywords:** Africa, Edible insects, Entomophagy, Local knowledge

## Abstract

**Background:**

Insects play an important role as a diet supplement in Burkina Faso, but the preferred insect species vary according to the phytogeographical zone, ethnic groups, and gender. The present study aims at documenting indigenous knowledge on edible insects in Burkina Faso.

**Methods:**

A structured ethno-sociological survey was conducted with 360 informants in nine villages located in two phytogeographical zones of Burkina Faso. Identification of the insects was done according to the classification of Scholtz. Chi-square tests and principal component analysis were performed to test for significant differences in edible insect species preferences among phytogeographical zones, villages, ethnic groups, and gender.

**Results:**

Edible insects were available at different times of the year. They were collected by hand picking, digging in the soil, and luring them into water traps. The edible insects collected were consumed fried, roasted, or grilled. All species were indifferently consumed by children, women, and men without regard to their ages. A total of seven edible insect species belonging to five orders were cited in the Sudanian zone of Burkina Faso. *Macrotermes subhyalinus* (Rambur), *Cirina butyrospermi* (Vuillet, 1911), *Kraussaria angulifera* (Krauss, 1877), *Gryllus campestris* (Linnaeus, 1758), and *Carbula marginella* (Thunberg) (35.66–8.47% of the citations) were most cited whereas *Rhynchophorus phoenicis* (Fabricius, 1801) and *Oryctes* sp*.* (3.41–0.27%) were least cited. *Cirina butyrospermi* was most cited in the South Sudanian zone, whereas *Macrotermes subhyalinus* and *Kraussaria angulifera* were most cited in the North Sudanian zone but were cited in all nine villages*. Cirina butyrospermi* was preferred by Bobo, Guin, Sambla, Senoufo, and Turka ethnic groups whereas *Macrotermes subhyalinus* was preferred by Fulani, Mossi, and Toussian ethnic groups. *Oryctes* sp*.* was cited only by the Toussian.

**Conclusion:**

A diversity of edible insects was consumed in both the South and North Sudanian zone of Burkina Faso with significant differences in species preferences according to phytogeographical zones, villages, ethnic groups, and gender.

## Background

The world consumption of meat was 41.2 kg/person/year in 2005 with a variation in developing countries from 82.1 to 13.3 kg in Sub-Saharan Africa [1]. From 2005 to 2050, world consumption of meat is predicted to increase 76% [1]. However, this increased consumption of meat has implications for habitat destruction, climate change, and human health [2, 3]. Alternative sources of animal proteins are highly needed. Among the sound alternatives, edible insects could occupy a prominent place. More than 2000 species of edible insects belonging to the orders of Coleoptera (beetles, often the larvae) (31%), Lepidoptera (caterpillars) (17%), Hymenoptera (wasps, bees, and ants) (15%), Orthoptera (crickets, grasshoppers, and locusts) (14%), Hemiptera (true bugs) (11%), Isoptera (termites) (3%), Odonata (dragonflies), Diptera (flies), and others (9%) have been worldwide reported by Jongema et al. [4]. The use of insects as an alternative source of protein has many advantages when compared with animals traditionally bred for food, as insects have a high feed conversion rate [5]. On average, 2 kg of food is needed to produce 1 kg of body mass in insects, whereas cattle require 8 kg of food to produce 1 kg of body mass [6]. Insects produce less greenhouse gases, use less water, and are less dependent on soil than conventional livestock [7, 8]. Unlike livestock, edible insects transmit only rather few known zoonotic diseases to humans [9]. The consumption of insects does not present risks of carcinogenic and cardiovascular diseases [10].

Although nutritional value varies from one species to another, edible insects are good sources of protein, amino acids, fats, vitamins, and minerals for human dietary needs [11]. Some caterpillars contain 50–60 g protein per 100 g dry weight, the palm weevil grubs 23–36 g, Orthoptera 41–91 g, ants 7–25 g, and termites 35–65 g [12–18]. The average fat contents range from 13.41% for Orthoptera (grasshoppers, crickets, locusts) to 33.40% for Coleoptera (beetles, grubs) and nearly 50% in Isoptera (termites) [11, 19, 20]. Analyses of nearly 100 species of edible insects have shown that the essential amino acid content is 10–30%, covering 35–50% of all types of amino acids, close to the amino acid consumption recommended by the World Health Organization and FAO [10, 19]. The fatty acids of insects are generally comparable to those of poultry and fish in terms of their degree of unsaturation [11]. Eggs, larvae, and pupae of honeybees have high amounts of vitamins A, B2, and C to the extent of 12.44 mg, 3.24 mg, and 10.25 mg/100 g, respectively [21]. Edible insects have the potential to provide specific micronutrients such as potassium, calcium, iron, and magnesium [12, 22, 23]. Termites have high iron contents [24, 25]. They contain more iron and calcium than beef, pork, and chicken [26]. Anthropo-entomophagy (eating of insects by humans) is practiced in 130 countries throughout the world by 3071 ethnic groups [27]. Edible species have been estimated at 679 in America, 524 in Africa, 349 in Asia, 152 in Australia, and 41 in Europe [28]. In Africa, there is a great variation in the number of edible species according the countries. Roulon-Doko [29] reported 96 edible species in the Central African Republic. In Nigeria, 23 edible species have been reported by Alamu et al. [12]. Riggi et al. [30] identified 29 arthropod species eaten in Benin. Ehounou et al. [31] described 9 edible species in the Ivory Coast. The most consumed insect species belong to Coleoptera, Hemiptera, Hymenoptera, Isoptera, Lepidoptera, and Orthoptera. A number of studies have reported differences between ethnic groups in the practice of entomophagy. Mofu-Gudur in Cameroon eat a number of grasshopper species (*Acorypha picta* (Krauss, 1877), *Acorypha glaucopsis* (Walker, 1870), *Acrida bicolor* (Thunberg, 1815)), which are not eaten by Hausas in Niger, and some insect species are consumed by Hausa people in Niger, which are not eaten by Mofu-Gudur [32, 33]. Riggi et al. [30] reported that the Waama ethnic group in the north of Benin preferentially consumed Coleoptera and Orthoptera in adult stage whereas Nagot and the Anii in the South preferred Orthoptera and Coleoptera only in the larval stage. In Burkina Faso, edible insects belonging to the orders of Orthoptera, Isoptera, and Lepidoptera are widely consumed [34]. However, available data on the distribution of the traditional knowledge regarding edible insects are very limited across the country, which is what led us to embark on this study.

## Methods

### Study area

**Fig. 1:**
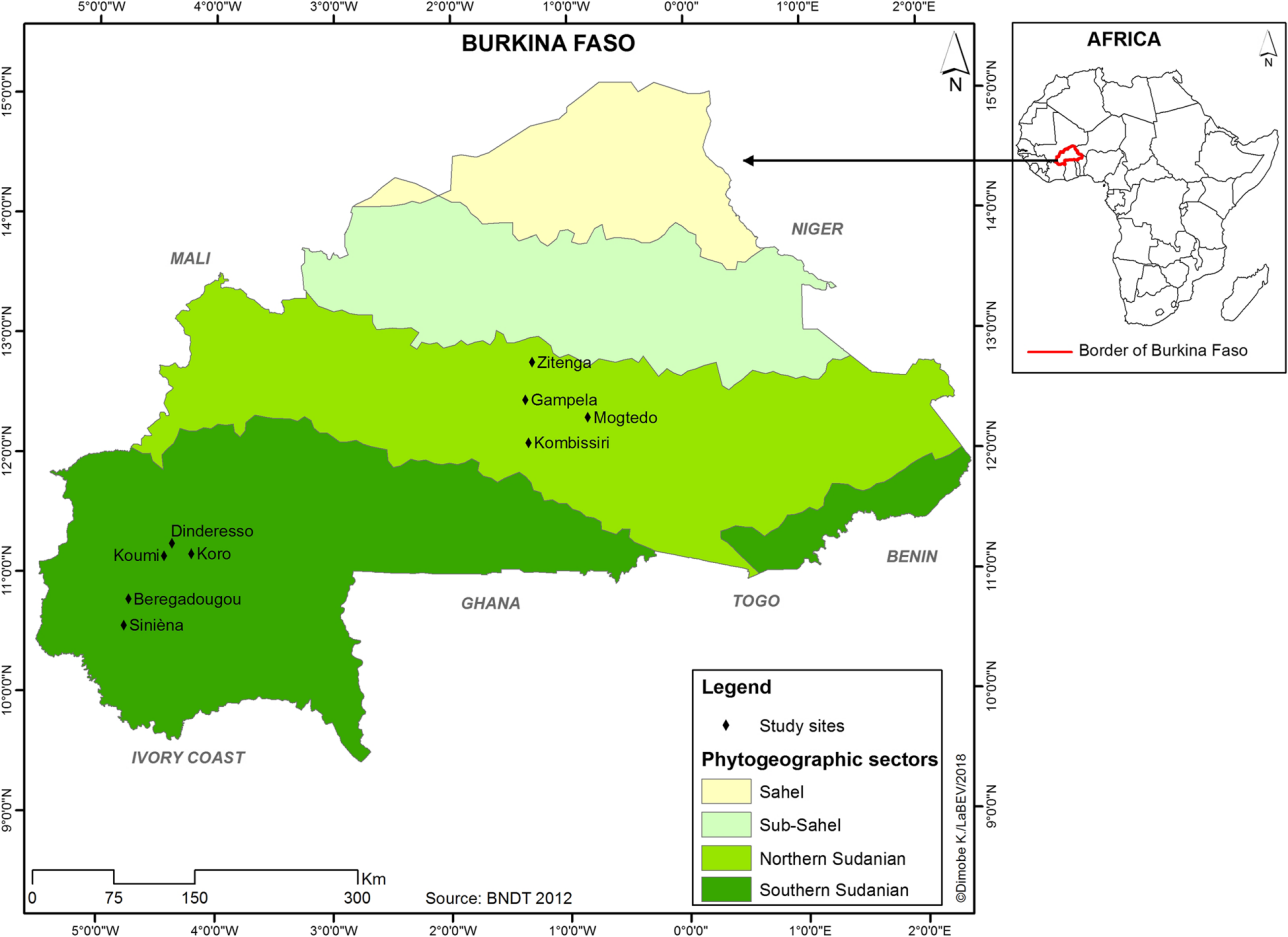
Burkina Faso with study villages indicated

The study was conducted from June to December 2015 and 2016 in nine villages across the Sudanian zone of Burkina Faso. They are Beregadougou, Dinderesso, Koro, Siniena, and Koumi located in the South Sudanian zone (9°–11°30′ N) and Gampela, Kombissiri, Mogtedo, and Zitenga in the North Sudanian zone (11°30′–14° N) (Fig. 1). In both study zones, the climate is dry tropical with a unimodal rainy season that lasts from May to October [35, 36]. Mean annual rainfall ranged from 600 to 900 mm in the North Sudanian zone and 900 to 1000 mm in the South Sudanian one [37]. The vegetation of the South Sudanian zone consists of a mosaic of savanna, dry forest, and patches of gallery forest and is characterized by Sudanian and Guinean species whereas the North Sudanian zone is dominated by savanna with annual growing grass, trees, and shrubs [35, 38, 39].

### Data collection

In each village, 40 informants were interviewed through individual semi-structured interviews. Members of all eight ethnic groups were interviewed in each village, when present. The 40 informants in each village included at least 30 natives of the zone and up to 10 non-natives without regarding their religious affiliation. Informants were between 15 and 65 years old. Informants were included in the questionnaire regardless of their education and occupation. A total of 186 men and 174 women were interviewed (Table 1). The questionnaire included the number of known edible insects, seasonal availability, stages of insects consumed, and modes of preparation. During interviews or at a suggested period, insect specimens were collected in bottles containing alcohol for identification using the Scholtz classification [40].

**Table 1:** Number of persons surveyed by ethnic group and village

Phytogeographical zone	Villages	Bobo	Fulani	Guin	Mossi	Sambla	Senoufo	Toussian	Turka	Total
South Sudanian	Beregadougou	0	0	13	2	3	7	2	13	40
South Sudanian	Dinderesso	17	0	0	6	0	5	12	0	40
South Sudanian	Koro	13	0	2	9	4	6	4	2	40
South Sudanian	Koumi	19	0	0	9	2	0	10	0	40
South Sudanian	Siniena	0	0	9	10	3	6	0	12	40
North Sudanian	Gampela	0	2	0	38	0	0	0	0	40
North Sudanian	Kombissiri	0	6	0	34	0	0	0	0	40
North Sudanian	Mogtedo	0	2	0	38	0	0	0	0	40
North Sudanian	Zitenga	0	0	0	40	0	0	0	0	40

### Data analysis

Chi-square analysis was used to determine whether there were statistically significant differences among zones, villages, ethnic group, and gender in knowledge and preference for edible insects. Statistical significance was tested at the 5% level. Principal component analysis (PCA) was used to explore patterns and variation in preferences among zones, villages, and ethnic groups. The used statistical software was XLSTAT-Premium 2016.

## Results

### Local knowledge extent on edible insects in Burkina Faso

**Fig. 2:**
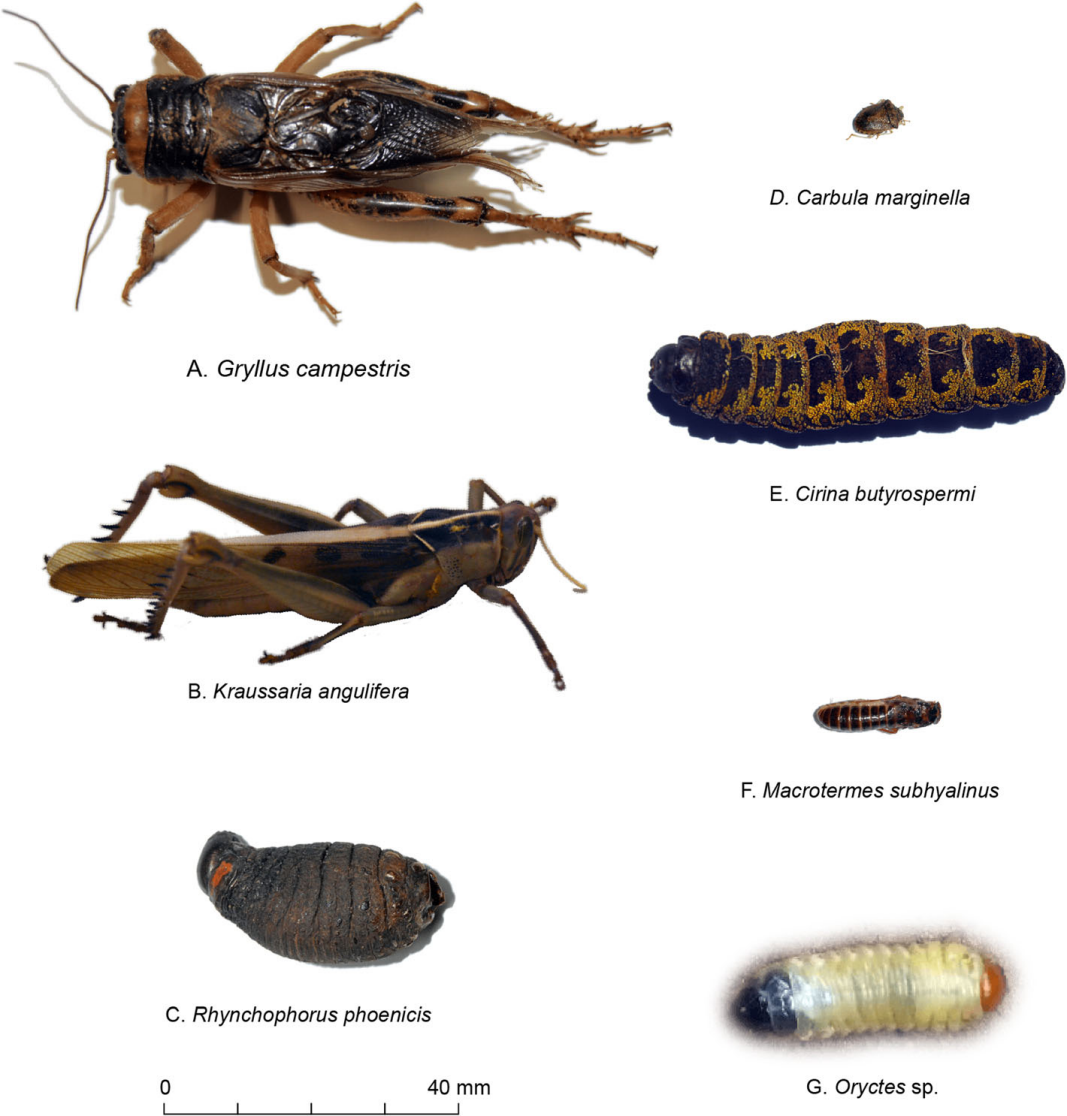
Edible insects across nine villages of Burkina Faso

**Fig. 3:**
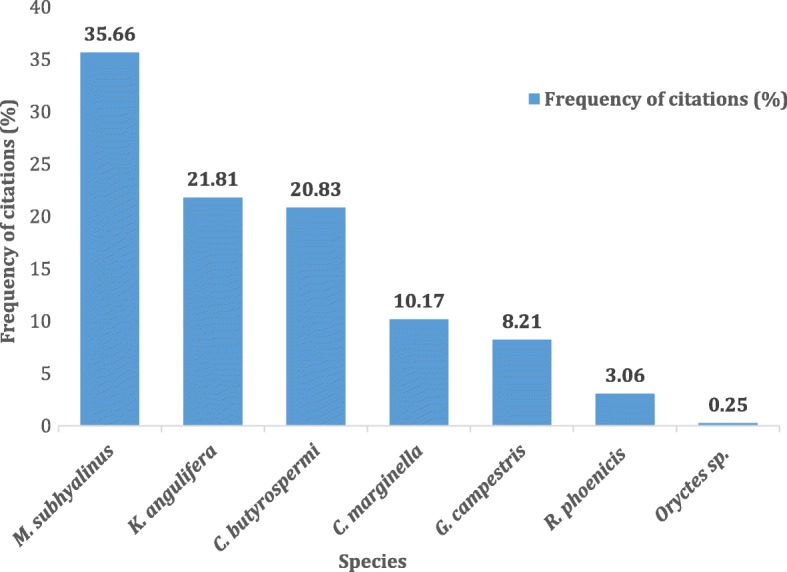
The frequency of citations of edible insects in nine villages of Burkina Faso

Seven edible insect species belonging to five orders were cited as consumed in the nine villages (Fig. 2). They were not consumed at the same stage of development: *Macrotermes subhyalinus*, *Kraussaria angulifera*, *Gryllus campestris*, and *Carbula marginella* were eaten at their adult stage whereas *Cirina butyrospermi*, *Rhynchophorus phoenicis*, and *Oryctes* sp. were eaten at the larval stage. *Macrotermes subhyalinus*, *Cirina butyrospermi*, and *Kraussaria angulifera* were the most cited whereas *Rhynchophorus phoenicis* and *Oryctes* sp*.* were the least cited (Fig. 3). 99.16% of informants ate at least one insect species. Significant differences were observed between zones, villages, ethnic groups, and gender.

### Distribution of cited edible insects according to phytogeographical zone

The citations of edible insect species varied according to phytogeographic zone (Table 2). Four species were cited in both the South and North Sudanian zones, among them *Cirina butyrospermi* and *Gryllus campestris* were most cited in the South Sudanian zone whereas *Kraussaria angulifera* and *Macrotermes subhyalinus* were most cited in the North Sudanian zone. *Rhynchophorus phoenicis* and *Oryctes* sp. were exclusively cited in the South Sudanian zone while *Carbula marginella* was exclusive to the North Sudanian zone.

**Table 2:** Percentage of citations of species by phytogeographical zone

Phytogeographical zones	*Macrotermes subhyalinus*	*Cirina butyrospermi*	*Kraussaria angulifera*	*Gryllus campestris*	*Carbula marginella*	*Rhynchophorus phoenicis*	*Oryctes* sp.
South Sudanian	62.5^a^	81.00^a^	21.00^a^	22.5^a^	0.00^a^	12.5^a^	1.00^a^
North Sudanian	85.00^b^	4.37^b^	64.37^b^	13.13^b^	38.75^b^	0.00^b^	0.00^a^

The citation percentages of the same column bearing different letters are significantly different (*p* < 0.05)

### Distribution of cited edible insects according to village

*Macrotermes subhyalinus* and *Kraussaria angulifera* were cited in all nine villages. *Gryllus campestris*, *Rhynchophorus phoenicis*, *and Oryctes* sp*.* were mentioned in eight, two, and one villages, respectively. *Cirina butyrospermi* and *Carbula marginella* were cited in three and four villages, respectively (Table 3).

**Table 3:** Percentage of citations of edible insect species by village

Phytogeographical zones	Villages	*Macrotermes subhyalinus*	*Cirina butyrospermi*	*Kraussaria angulifera*	*Gryllus campestris*	*Carbula marginella*	*Rhynchophorus phoenicis*	*Oryctes* sp.
South Sudanian	Beregadougou	50.00	95.00	17.50	7.50	0.00	30.00	0.00
South Sudanian	Dinderesso	85.00	85.00	35.00	50.00	0.00	0.00	5.00
South Sudanian	Koro	62.50	75.00	10.00	10.00	0.00	0.00	0.00
South Sudanian	Koumi	67.50	77.50	25.00	40.00	0.00	0.00	0.00
South Sudanian	Siniena	47.50	72.50	20.00	5.00	0.00	32.50	0.00
North Sudanian	Gampela	72.50	0.00	62.50	32.50	25.00	12.50	0.00
North Sudanian	Kombissiri	92.50	17.50	65.00	17.50	2.50	0.00	0.00
North Sudanian	Mogtedo	100.00	0.00	60.00	0.00	75.00	0.00	0.00
North Sudanian	Zitenga	75.00	0.00	70.00	2.50	52.50	0.00	0.00

In the North Sudanian zone, the consumption of the species *Cirina butyrospermi*, *Carbula marginella*, *Gryllus campestris*, and *Macrotermes subhyalinus* was significantly different among villages ((*X*^2^ = 21.96; df = 3; *p* < 0.0001), (*X*^2^ = 50.66; df = 3; *p* < 0.0001), (*X*^2^ = 23.84; df = 3; *p* < 0.0001), (*X*^2^ = 16.86; df = 3; *p* = 0.0008)). There was no difference in the consumption of *Kraussaria angulifera.*

In the South Sudanian zone, there was a significant difference in the consumption of *Gryllus campestris*, *Rhynchophorus phoenicis*, and *Macrotermes subhyalinus* ((*X*^2^ = 40.14; df = 4; *p* < 0.0001), (*X*^2^ = 42.97; df = 4; *p* < 0.0001), (*X*^2^ = 15.57; df = 4; *p* = 0.0036)). No significant differences were found for *Kraussaria angulifera*, *Cirina butyrospermi*, and *Oryctes* sp.

**Fig. 4:**
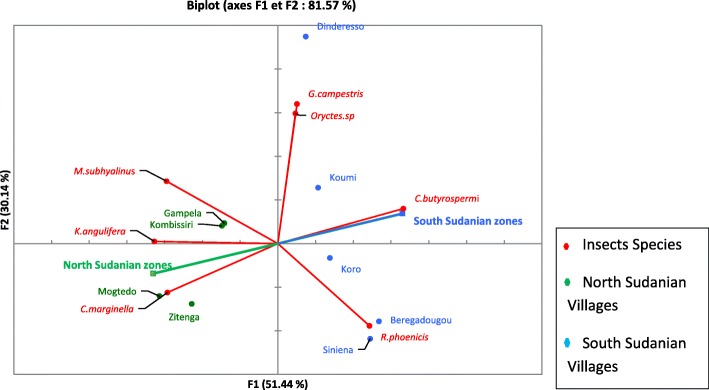
Principal component analysis (PCA) of the preference for consumption of edible insects in nine villages and two phytogeographical zones of Burkina Faso

The principal component analysis clearly showed that people from different villages had different preferences (Fig. 4). *Kraussaria angulifera* and *Carbula marginella* were the most cited in Mogtedo and Zitenga, *Macrotermes subhyalinus* was the most cited in Gampela and Kombissiri. *Gryllus campestris* and *Oryctes* sp*.* were the most cited in Dinderesso, *Cirina butyrospermi* was the most cited in Koumi and Koro, and *Rhynchophorus phoenicis* was the most cited in Beregadougou and Siniena.

### Knowledge of edible insects among ethnic groups

*Macrotermes subhyalinus*, *Cirina butyrospermi*, and *Gryllus campestris* were mentioned as edible insects by all nine ethnic groups whereas *Kraussaria angulifera*, *Rhynchophorus phoenicis*, *Carbula marginella*, and *Oryctes* sp. were cited by seven, five, two, and one ethnic groups, respectively (Table 4).

**Table 4:** Percentage of citations of edible insect species by ethnic group

Ethnic groups	*Macrotermes subhyalinus*	*Cirina butyrospermi*	*Kraussaria angulifera*	*Gryllus campestris*	*Carbula marginella*	*Rhynchophorus phoenicis*	*Oryctes* sp.
Bobo	63.26	81.63	16.32	30.61	0.00	0.00	0.00
Fulani	85.71	7.14	35.71	0.00	28.57	0.00	0.00
Guin	41.67	79.16	20.83	8.33	0.00	29.16	0.00
Mossi	79.67	18.13	57.14	15.93	31.87	2.20	0.00
Sambla	50.00	91.67	0.00	16.67	0.00	16.66	0.00
Senoufo	66.67	91.67	8.33	20.83	0.00	16.67	0.00
Toussian	96.43	71.43	57.14	39.28	0.00	0.00	7.14
Turka	51.85	85.18	22.22	7.41	0.00	29.63	0.00

In the North Sudanian zone, there was no significant difference in the consumption of *Cirina butyrospermi*, *Gryllus campestris*, *Carbula marginella*, and *Macrotermes subhyalinus* among ethnic groups, whereas a significant difference (*X*^2^ = 5.49; df = 1; *p* = 0.019) was found for *Kraussaria angulifera* which was highly preferred by the Mossi.

**Fig. 5:**
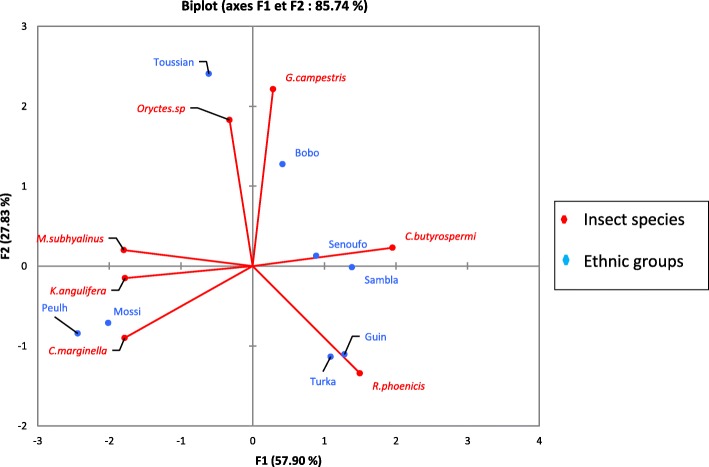
Principal component analysis (PCA) of the preference for consumption of edible insects of ethnic groups in two phytogeographical zones of Burkina Faso

In the South Sudanian zone, the consumption of *Gryllus campestris*, *Rhynchophorus phoenicis*, *Macrotermes subhyalinus*, and *Cirina butyrospermi* was significantly different among ethnic groups ((*X*^2^ = 12.93; df = 6; *p* = 0.04), (*X*^2^ = 24.97; df = 6; *p* = 0.0003), (*X*^2^ = 20.76; df = 6; *p* = 0.002), (*X*^2^ = 16.63; df = 6; *p* = 0.038)). That of *Kraussaria angulifera* and *Oryctes* sp. was not associated with the ethnic group. The principal component analysis clearly showed edible insect preference according to ethnic group (Fig. 5). Bobo, Guin, Sambla, Senoufo, and Turka ethnic groups preferentially mentioned *Cirina butyrospermi* while *Macrotermes subhyalinus* was the most mentioned by Fulani, Mossi, and Toussian ethnic groups. *Oryctes* sp. was cited only by the Toussian ethnic group.

### Knowledge of edible insects according to gender

**Fig. 6:**
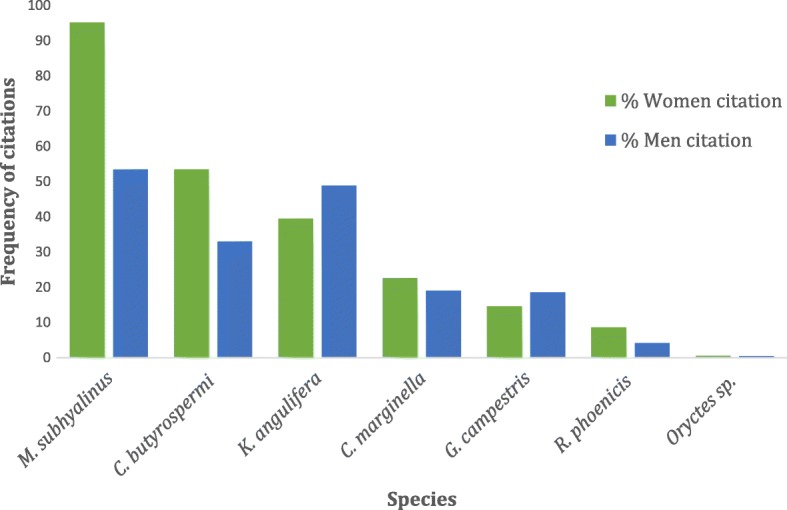
Frequency of citations according to gender of nine edible insects of Burkina Faso

The preferences of edible insects varied according to gender (Fig. 6). *Cirina butyrospermi*, *Macrotermes subhyalinus*, *Rhynchophorus phoenicis*, and *Carbula marginella* were most cited by women whereas *Gryllus campestris* and *Kraussaria angulifera* were preferentially mentioned by men. *Oryctes* sp. was cited by both men and women in the same proportions.

### Seasonal occurrence, collection, and forms of consumption of edible insects

The period of availability, collection techniques, and forms of consumption differed from one species to another (Table 5). The seasonal occurrence was related to seasonal conditions. *Macrotermes subhyalinus*, *Cirina butyrospermi*, and *Oryctes* sp. occurred during the rainy season whereas *Carbula marginella* (Thunberg), *Gryllus campestris*, *Kraussaria angulifera*, and *Rhynchophorus phoenicis* occurred during the dry season. Collection techniques were specific to species. Children were involved in the collection of all species. Women specifically picked up *Macrotermes subhyalinus*, *Cirina butyrospermi*, and *Carbula marginella.* Men preferentially harvested *Gryllus campestris* and *Kraussaria angulifera.* Both women and men were involved in the collection of *Rhynchophorus phoenicis.* The consumption form common to all species is fried, followed by roasted (*Macrotermes subhyalinus*, *Rhynchophorus phoenicis*, and *Oryctes* sp.) and grilled (*Kraussaria angulifera* and *Gryllus campestris*)*.* All species were indifferently consumed by children, women, and men without regard to their ages.

**Table 5:** Seasonal occurrence, collection, and forms of consumption of edible insects

Scientific name	Orders	Common name	Seasonal availability	Consumption stage	Methods of collection	Person who collect	Forms of consumption
*Macrotermes subhyalinus*	Isoptera	Winged termites	June–July	Adult	Trapped in a large bowl of water near the light source	Children, women	Fried, roasted
*Cirina butyrospermi*	Lepidoptera	Caterpillar	June–August	Larva	Picked up under the plant	Children, women	Fried, ingredients in sauce
*Kraussaria angulifera*	Orthoptera	Grasshopper	November–January	Adult	Harvested by hand or with a stick very early in the morning	Men, children	Fried, grilled
*Gryllus campestris*	Orthoptera	Field cricket	September–October	Adult	Hunted by digging them out from their burrows	Men, children	Fried, grilled
*Carbula marginella*	Hemiptera	Beetle	October–January	Adult	Picked up under the cave holes and millet	Children, women	Fried
*Rhynchophorus phoenicis*	Coleoptera	Palm weevil	December–May	Larva	Picked up inside the infested host plant	Children, men, women	Fried, roasted
*Oryctes* sp.	Coleoptera	–	June–August	Larva	Picked in the cow dung	Children, women	Fried, roasted

## Discussion

### Distribution of cited edible insects according to phytogeographical zone and villages

The number of edible insects (seven species) in the current study was less than those reported from other African countries. Takeda [41] reported 21 species consumed by the Ngandu people in the Democratic Republic of Congo. Malaisse [42] inventoried 30 edible species in northern Zambia, RDC and northeastern Zimbabwe. Obopile and Seeletso [43] identified 27 edible insects in Botswana. Twenty-two (22) insect species belonging to six different orders have been recorded with potential for consumption among the three major ethnic groups (Yoruba, Hausa, and Ibo) in Nigeria [12].

*Kraussaria angulifera*, *Cirina butyrospermi*, *Gryllus campestris*, *Macrotermes subhyalinus*, *Oryctes* sp., and *Rhynchophorus phoenicis* have already been reported as edible insect species in different parts of Africa. However, to the best of our knowledge, this study is the first report on *Carbula marginella* as an edible insect. No prohibition regarding any species has been noted during the survey in contrast to that reported in Nigeria [44].

Each species develops only under specific climatic conditions. *Cirina butyrospermi* and *Oryctes* sp. reproduce exclusively in the south Sudanian zone under specific rainfall (900 to 1000 mm) and humidity conditions [37]. *Rhynchophorus phoenicis* was available in the South Sudanian zone. *Cirina butyrospermi* is dried and marketed throughout the country while the consumption of *Oryctes* sp. and *Rhynchophorus phoenicis* was restricted to the South Sudanian zone. *Carbula marginella* was found only in the northern Sudanian area. Its consumption is restricted to its production zone due to the absence of a trade system. *Gryllus campestris* and *Macrotermes subhyalinus* were available in both South and North Sudanian zones where they are well consumed. *Macrotermes subhyalinus* is marketed throughout the country.

### Knowledge of edible insects among ethnic groups

In addition to the availability, another parameter which influences species edibility is ethnic preference. All the eight ethnic groups were entomophagous. However, there are considerable differences in preferences among them. Similar results were reported in Benin where specific preferences were observed among several ethnic groups. In the South of Benin, the most consumed insects across different localities were the larvae of *Oryctes* sp. and *Rhynchophorus phoenicis*. On the contrary, in the North, an assemblage of more varied insect species was consumed across different localities [30]. Riggi et al. [30] linked the richness of edible insect species in the North of Benin to the poverty and the unreliable productivity of agriculture in this region. Observation on the distribution and consumption of edible insects in Nigeria revealed that the practice of entomophagy is common in the humid forest, derived savanna, and some parts of Southern Guinea Savanna agro-ecological zones of the country [12]. In Burkina Faso, the high diversity of edible insects was observed in the South Sudanian zone which is known as the agricultural belt of the country. Diversity of consumed insects seems therefore to be more linked to species availability and people’s alimentary habits and culture. These factors could explain why the Waama ethnic group in Benin only eat the adult and not the larvae of Lepidoptera, such as *Cirina butyrospermi* [30]. In the same way, the Mofu-Gudur in Cameroon eat a number of grasshopper species (*Acorypha picta*, *A*. *glaucopsis*, *Acrida bicolor*, *Oedaleus senegalensis* (Krauss, 1877), *Pyrgomorpha cognate* (Krauss, 1877), *Truxalis johnstoni* (Dirsh, 1951)), which are not eaten by the Hausa in Niger, and vice versa (*Humbe tenuicornis* (Schaum, 1853)) [32, 33]. Change in alimentary habit and culture could also explain why Mossi and Fulani ethnic migrants in the South Sudanese zone eat *Cirina butyrospermi* whereas members of the same ethnic groups in the North Sudanian zone do not. One consequence of such differences in preferred species, as explained by Meyer-Rochow [45], is that pressure on a resource is distributed across a range of species and in this way it helps to avoid an overexploitation of the resource. In the same line, species availability, alimentary habit, and culture could also explain the restriction of the consumption of *Oryctes* sp. and *Carbula marginella* to specific zones and ethnic groups of Burkina Faso.

### Knowledge of edible insects according to gender

Women and children are most involved in insect collection. Women are the main actors in the collection and sale of edible insects. In southern Zimbabwe, the collection, processing, and marketing of mopane caterpillars (*Imbrasia belina* (Westwood, 1849)) were traditionally practiced by women [46, 47]. This activity generates income for these women and their families. These incomes are used for food, child rearing, and other family expenses [48, 49].

### Seasonal occurrence, collection, and forms of consumption of edible insects

As reported, the seasonal availability is mostly influenced by environmental factors such as temperature and relative humidity. On this basis, two groups of species can be distinguished: rainy season species (*Macrotermes subhyalinus*, *Cirina butyrospermi*, and *Oryctes* sp.) and dry season species (*Carbula marginella*, *Gryllus campestris*, *Kraussaria angulifera*, and *Rhynchophorus phoenicis*). In addition to these factors, host availability is another key factor for some species. Thus, the availability of *Cirina butyrospermi* is close to the availability of its host shea tree. Women and children are once again the main edible insect collectors. The collection methods can be grouped in easy (by hand) and hard collecting methods (digging in the soil). Species can also be classified in income-generating (*Macrotermes subhyalinus*, *Cirina butyrospermi*, *Carbula marginella*) and non-income-generating species (*Gryllus campestris*, *Rhynchophorus phoenicis*, *Kraussaria angulifera*, and *Oryctes* sp.). Women are mostly involved in easy-to-collect and income-generating species. Children and men are dominant collectors when physical effort is required. There is a diversity in the methods of preparation. Hongbété and Kindossi [50] reported that the edible insects were sun-dried, fried, and smoked or roasted. In some cases, edible insects are used as condiment in slimy and vegetable sauces [50]. Chakravorty et al. [51] reported also that short-horned grasshoppers (Acrididae) are fried.

Riggi et al. [52] reported that in the Northern Benin, children between 5 and 15 years of age chased in groups edible insects that they collected in a jar, cooked in a pan with shea butter, or grilled directly on charcoal. The larva of *Oryctes monoceros* is boiled, smoked, or fried [53]. The Bambaras in Mali and Burkina Faso fried *Cirina forda* in shea tree [54]. *R*. *phoenicis* is often grilled or fried on charred coals [55].

## Conclusion

Our survey revealed seven edible insect species in the nine villages studied. The knowledge of edible species varied from one locality to another and between ethnic groups. *Cirina butyrospermi*, *Oryctes* sp., *Rhynchophorus phoenicis*, and *Gryllus campestris* were the most cited in the South Sudanian zone, whereas *Macrotermes subhyalinus*, *Carbula marginella*, and *Kraussaria angulifera* were most cited in the North Sudanian zone. Bobo, Guin, Sambla, Senoufo, and Turka ethnic groups mentioned *Cirina butyrospermi* as the preferred species, but Fulani, Mossi, and Toussian preferred *Macrotermes subhyalinus*. *Oryctes* sp*.* was cited only by the Toussian ethnic group. As insects are used and appreciated as food in Burkina Faso, there is considerable potential to further develop this commodity.
